# pH-/Temperature-Triggered Gel Transition of Hyperbranched PEI-g-PDMAEMA as a Dual-Responsive Inhibitor for Clay Hydration Control

**DOI:** 10.3390/gels12090830

**Published:** 2026-09-10

**Authors:** Ming Zhong, Yang Xiong

**Affiliations:** 1 School of Energy and Resources, China University of Geosciences (Beijing), Beijing 100083, China; 2 School of Petroleum Engineering, Yangtze University, Wuhan 430100, China

**Keywords:** stimuli-responsive polymer, hyperbranched polyethyleneimine, clay hydration inhibition, lower critical solution temperature, hydrophobic association, zeta potential

## Abstract

To mitigate clay hydration and wellbore instability during deepwater drilling, a pH/temperature dual-responsive graft copolymer, hyperbranched polyethylenimine-g-poly(2-(dimethylamino)ethyl methacrylate) (HPEI-g-PDMAEMA), was designed and synthesized via free radical polymerization. Optimized synthesis at an HPEI/DMAEMA mass ratio of 1:2 with 2.4% AIBN at 70 °C for 10 h yielded a grafting ratio of 35.2% and a molecular weight of 84.3 kDa. The copolymer exhibits a tunable lower critical solution temperature (LCST) of approximately 48 °C at pH 8, decreasing with increasing pH due to tertiary amine deprotonation. Zeta potential measurements confirm that the polymer retains a positive charge (+5 mV at pH 8) under weakly alkaline conditions, enabling strong electrostatic anchoring onto negatively charged clay surfaces. Above the LCST, dynamic light scattering reveals a sharp increase in hydrodynamic diameter from ~30 nm to >200 nm, confirming a hydrophilic-to-hydrophobic transition of PDMAEMA segments that drives the formation of a hydrophobically associated gel barrier. This thermally triggered gelation is fully reversible, as evidenced by repeated heating–cooling cycles with almost complete transmittance recovery. The gel barrier drastically reduces water uptake, with inhibition performance against clay swelling at 60 °C being 18.5 percentage points higher than that at 25 °C. Hot-rolling tests demonstrate that with only 1.5 wt% inhibitor, shale recovery reaches 94.1% at 150 °C (8.8 percentage points higher than unmodified HPEI) and remains above 60% even in 20 wt% CaCl_2_ or MgCl_2_ brines, highlighting exceptional resistance to divalent cations. Water contact angle on treated clay surfaces increases from 18.5° to 52.6°, confirming effective surface hydrophobization. This work provides a molecular-level gel-engineering strategy where pH governs electrostatic anchoring and temperature triggers reversible hydrophobic gelation, enabling on-demand switching of clay wettability and hydration resistance under high-temperature, high-salinity conditions.

## 1. Introduction

The reliability and cost-effectiveness of hydrocarbon extraction increasingly depend on drilling fluid technology, where even marginal improvements in shale inhibition can translate into substantial operational savings. Among many critical factors, drilling fluid is often called the “lifeblood” of drilling operations, and its performance directly determines the success or failure of the entire operation. Generally, drilling fluids can be classified into water-based drilling fluids, oil-based drilling fluids, and synthetic-based drilling fluids [1,2,3,4,5]. Among them, water-based drilling fluids have advantages such as cost-effectiveness, low toxicity, and excellent rheological properties [6,7,8,9,10]. However, when drilling highly reactive shale formations, clay minerals readily undergo hydration swelling and dispersion, leading to wellbore instability, collapse, and even well abandonment [11,12,13,14,15]. While traditional inhibitors (such as KCl, polyethylene glycol, polyamines, etc.) offer certain inhibition effects, they suffer from high dosage requirements, poor environmental friendliness, or performance degradation at elevated temperatures [16,17,18,19,20]. In recent years, polymer-based inhibitors have received wide attention due to their highly designable molecular structures and tunable inhibition performance [16,21,22,23,24]. Li et al. [25] used a polysaccharide derivative (alginate peptide) as a raw material, modified it with N, N-dibutyl-1,3-propanediamine and bromoalkane, and prepared a novel, environmentally friendly inhibitor, HBM-T, which significantly improved the sealing and inhibition performance of water-based drilling fluids in shale formations. Li et al. [26] developed a nanocomposite N-AMPA as the main agent and constructed a solids-free drilling fluid system with excellent temperature resistance, enabling temperature-controlled plugging in deep-water water-based drilling fluids and effectively enhancing wellbore stability. Although the above thermosensitive materials have achieved positive progress, the structural design of the polymer backbone is equally crucial.

From a colloid and interface perspective, clay hydration is fundamentally controlled by the adsorption of polymers onto clay surfaces. HPEI possesses numerous amine groups and can firmly anchor onto clay surfaces via electrostatic adsorption, exhibiting strong shale inhibition capability, and thus has attracted extensive attention from researchers in oil and gas fields. However, the inhibitory effect of HPEI decreases at high temperatures, lacking temperature-adaptive ability, so modification is often used to enhance inhibition performance [23,27,28,29,30]. Stimuli-responsive polymers offer an attractive solution by enabling on-demand switching of surface properties. In particular, polymers exhibiting an LCST undergo a hydrophilic-to-hydrophobic transition upon heating, which can be harnessed to form a water-blocking film on clay surfaces [31,32,33,34,35]. Poly(2-(dimethylamino)ethyl methacrylate) (PDMAEMA) is a well-known pH/Temperature dual-responsive polymer: its tertiary amine groups confer pH-dependent protonation, and its LCST can be tuned between approximately 40 °C and 60 °C depending on the pH and ionic strength [36]. The cationic nature of PDMAEMA is key to its LCST behavior: its tertiary amines (pK_a_ ≈ 7.0–7.8) become partially deprotonated in the alkaline drilling fluid environment (pH 8–9), and heating above the LCST disrupts hydrogen bonding with water, driving a hydrophilic-to-hydrophobic collapse that forms a water-blocking barrier on clay surfaces [37,38,39]. Grafting PDMAEMA onto a hyperbranched backbone could combine the strong anchoring capability of HPEI with the thermo-responsive hydrophobicity of PDMAEMA, leading to a “low-temperature dispersion, high-temperature enhancement” inhibition behavior. To date, however, such a dual-responsive graft copolymer has not been reported for clay hydration control. Recent studies have explored various thermoresponsive shale inhibitors. For instance, Hu et al. [40] reported an HPEI-PO-PNIPAM terpolymer, in which PNIPAM provides thermosensitive hydrophobicity, and PO acts as a permanent hydrophobic monomer. However, this system has limitations: (i) the LCST of PNIPAM (~32 °C) is close to or below surface temperatures, causing premature collapse and reduced effective concentration before reaching the downhole zone; (ii) PNIPAM mainly adsorbs via hydrogen bonds through amide groups, which are weak and prone to desorption under high-temperature, high-salinity conditions.

To overcome these issues, we designed a binary HPEI-g-PDMAEMA graft copolymer with the following innovations: (a) a tailored LCST window—the LCST of DMAEMA in alkaline drilling fluid (pH 8–9) is ≈45–48 °C, which is well above surface temperature but below common downhole temperatures, enabling “surface-dispersed, downhole-activated” smart inhibition; (b) strong electrostatic anchoring—the HPEI backbone remains partially protonated under weak alkaline conditions, providing strong electrostatic attraction to negatively charged clay surfaces, making our method far more robust than hydrogen bonding; (c) synthetic simplicity—grafting a single monomer avoids the complex synthesis of terpolymers and ensures good water solubility.

## 2. Results and Discussion

### 2.1. Optimization of Synthesis Conditions

#### 2.1.1. Effect of Feed Ratio

The reaction temperature was fixed at 70 °C, with a reaction time of 10 h and an initiator dosage of 0.12 g (2.4% relative to HPEI). The effect of the HPEI/DMAEMA mass ratio on product performance was investigated. As shown in Table 1, the LCST of the copolymers decreased with increasing DMAEMA content (from 5 °C to 38 °C), confirming enhanced hydrophobicity. However, the decline in recovery at feed ratios ≥ 1:2.5 cannot be solely explained by LCST depression. More critically, the room-temperature transmittance at 25 °C dropped sharply from 94.8% (1:2) to 78.4% (1:2.5) and further to 62.1% (1:3). This quantitative evidence confirms that the 1:2.5 and 1:3 products already underwent microphase separation or partial precipitation at ambient temperature due to excessive hydrophobic PDMAEMA content. Such poor water dispersibility at the surface limited the effective polymer concentration available for adsorption onto clay particles before even reaching the downhole zone, thereby compromising the inhibition performance despite the higher grafting ratio. This may be because excessively high DMAEMA content resulted in excessive hydrophobicity, affecting the dispersibility of the polymer in water, which in turn compromised inhibition performance. Therefore, HPEI: DMAEMA = 1:2 was chosen as the optimal feed ratio. The decline in recovery at feed ratios above 1:2 (1:2.5 and 1:3) can be rationalized by steric hindrance effects. Excessively long or densely grafted PDMAEMA side chains adopt extended conformations in aqueous solutions, which may physically shield the amine groups on the HPEI backbone from approaching the clay surface. Such steric occlusion reduces the effective electrostatic anchoring between the polymer and clay particles. Moreover, overly dense grafts restrict the conformational flexibility required for the polymer to adapt to the clay surface topology, thereby weakening the inhibition efficiency. This interpretation is consistent with the observed opalescence at a 1:3 feed ratio, indicating reduced aqueous dispersibility due to excessive hydrophobic content.

From a conformational perspective, densely grafted PDMAEMA side chains adopt extended coil conformations that physically shield the amine anchoring groups on the HPEI backbone from approaching the clay surface, reducing electrostatic anchoring density. The steric congestion also restricts chain flexibility, limiting the polymer’s ability to adapt to the clay surface topology during adsorption. Consequently, the inhibitor adsorbs in a looser manner, reducing surface coverage and allowing water molecules to penetrate into interlayer spaces. This steric effect is particularly pronounced at feed ratios ≥ 1:2.5, where dense grafts compromise the accessibility of active anchoring groups, leading to the observed decline in recovery despite higher grafting ratios.

#### 2.1.2. Effect of AIBN Dosage

With other conditions fixed, the effect of AIBN dosage on product performance was examined (Table 2). When the AIBN dosage was too low (1.0% of HPEI mass), the product molecular weight became too high (125,243), and a small amount of gel was observed during dialysis, likely because the radical concentration was too low, leading to insufficient chain termination. When the AIBN dosage was too high (4.0%), the molecular weight decreased to 64,281, and the inhibition performance significantly declined. At an AIBN dosage of 2.4%, the product had a moderate molecular weight (84,298), no gelation, and the highest 150 °C recovery of 94.1%. Therefore, 2.4% was selected. The optimal AIBN dosage (2.4 wt%) was selected based on a comprehensive evaluation of three criteria: (i) product molecular weight within a moderate range to ensure good water dispersibility; (ii) absence of gelation during dialysis, which indicates sufficient chain termination and minimal crosslinking; and (iii) the highest hot-rolling recovery at 150 °C (94.1%). Although higher AIBN dosages (≥3.0%) yielded lower molecular weights, the corresponding recovery decreased, likely due to excessive chain scission, which would have reduced the anchoring efficiency of the polymer on clay surfaces.

#### 2.1.3. Effect of Reaction Temperature

The reaction time was fixed at 10 h, and the AIBN dosage was 2.4%. The effect of temperature on product performance is shown in Table 3. As the temperature increased from 60 °C to 70 °C, the grafting ratio increased from 28.6% to 35.2%, and the 150 °C hot-rolling recovery rate increased from 89.2% to 94.1%. When the temperature was further raised to 75 °C and 80 °C, although the grafting ratio increased slightly, gelation appeared in the product, molecular weight increased significantly, and recovery decreased to 92.4% and 88.6%, respectively. This is because excessive temperature induces side reactions, leading to polymer crosslinking, which affects its dispersibility and adsorption ability in water. Therefore, 70 °C was selected as the optimal reaction temperature. The occurrence of gelation at 80 °C, despite a slight increase in grafting ratio, is ascribed to conformational changes in the cationic HPEI backbone at elevated temperatures. As temperature increases, the hydrogen-bonding interactions between HPEI amine groups and the solvent weaken, promoting interchain hydrophobic association and aggregation. Additionally, the decreased chain mobility at higher temperatures favors intermolecular crosslinking rather than intramolecular grafting, resulting in a three-dimensional network. This gelation not only reduces the solubility of the product but also compromises its ability to adsorb uniformly onto clay surfaces, as reflected by the decreased recovery at 80 °C.

#### 2.1.4. Effect of Reaction Time

With other conditions fixed, the effect of reaction time was examined (Table 4). When reaction time increased from 6 h to 10 h, the grafting ratio increased from 24.5% to 35.2%, and recovery increased from 86.5% to 94.1%. Further extending the reaction time to 12 h and 14 h resulted in a slower increase in grafting ratio, a continuous increase in molecular weight, slight gelation at 14 h, and a slight decrease in recovery. Considering reaction efficiency and product performance, the optimal reaction time was determined as 10 h.

#### 2.1.5. Mechanistic Considerations on Copolymerization

The trends observed in the optimization of synthesis conditions can be rationalized from the perspective of free radical copolymerization kinetics and the cationic nature of DMAEMA. The grafting ratio increased with increasing DMAEMA feed ratio and reaction time (Table 1 and Table 4), consistent with conventional radical chain-growth behavior. However, the decline in recovery at feed ratios above 1:2 is attributed to the excessive hydrophobicity and steric hindrance of densely grafted PDMAEMA chains, which restrict conformational flexibility for effective clay anchoring. AIBN dosage (Table 2) governs the balance between chain initiation and termination: insufficient radical flux at a low dosage prolongs chain growth and induces gelation, whereas excessive radicals at a high dosage promote chain scission and reduce anchoring efficiency. Temperature (Table 3) exerts dual effects—elevated temperature enhances initiation and propagation up to 70 °C, but a further increase weakens hydrogen bonding between HPEI amine groups and the solvent, promoting interchain hydrophobic association and crosslinking, which compromise product solubility and adsorption performance. Additionally, the tertiary amine groups of DMAEMA introduce electrostatic repulsion with the protonated amines of HPEI, influencing monomer accessibility and grafting homogeneity, while the increasing viscosity of the reaction medium may further retard monomer diffusion as molecular weight increases. These kinetic, electrostatic, and viscosity-related factors collectively account for the observed dependence of grafting ratio and product performance on the synthesis parameters.

### 2.2. Characterization of Polymer Molecular Structures

Based on the grafting ratio of 35.2% determined by gravimetric analysis, the number of PDMAEMA side chains per HPEI molecule is roughly estimated to be approximately 3, corresponding to a grafting density of about 0.2–0.3 side chains per 100 HPEI repeat units, assuming an average degree of polymerization of 40–50 for the grafted chains. This gravimetric value is used primarily for comparing samples synthesized under different conditions.

**Figure 1:**
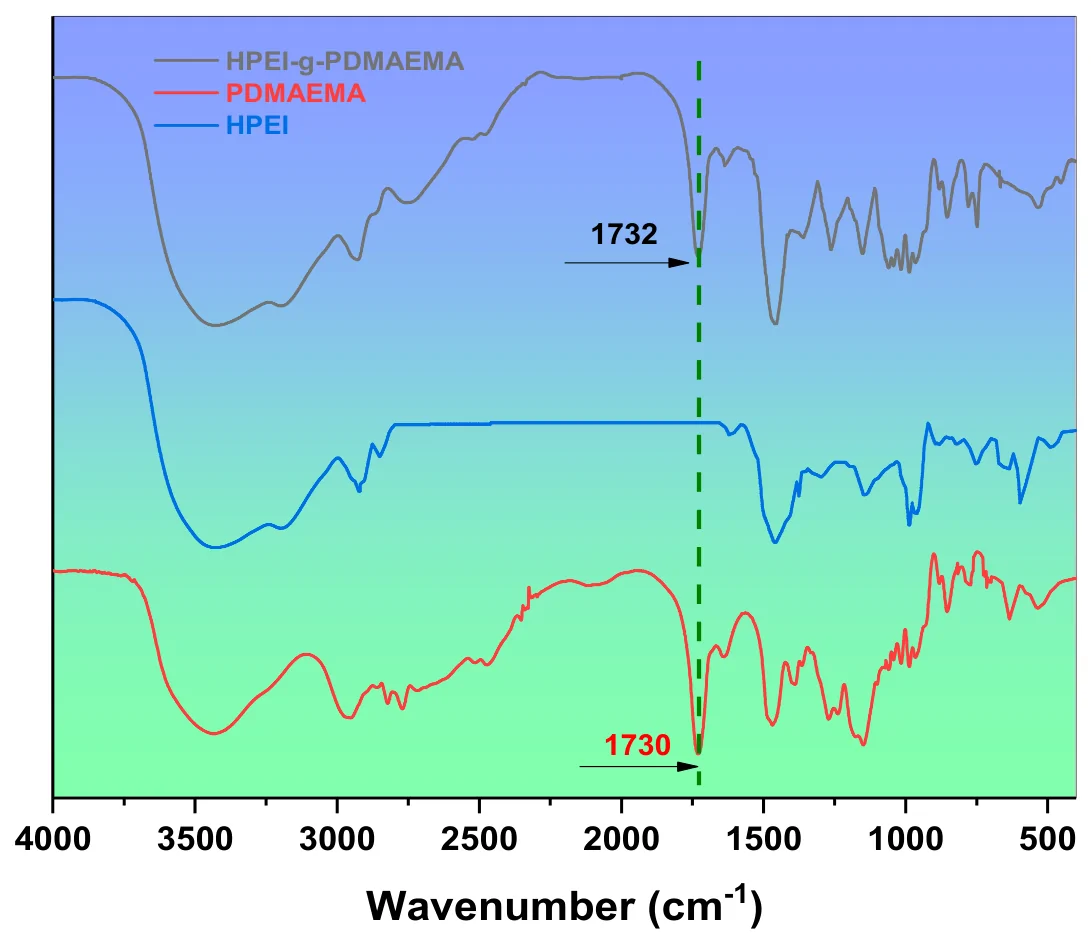
Infrared spectrum of HPEI, PDMAEMA, and HPEI-g-PDMAEMA.

Figure 1 shows the FTIR spectrum of HPEI, PDMAEMA, and HPEI-g-PDMAEMA. A broad and strong absorption band at 3428 cm^−1^ is attributed to N–H stretching vibrations of primary and secondary amines in the polymer HPEI backbone, while the bands at 2930 cm^−1^ correspond to the asymmetric C-H stretching of methylene groups. A sharp and intense absorption peak at 1730 cm^−1^, which is absent in pure HPEI but present in both pure PDMAEMA and the grafted copolymer, is assigned to the ester carbonyl (C=O) stretching vibration from the PDMAEMA segments, providing direct evidence of successful grafting. In addition, the absorptions at 1263 cm^−1^ and 1151 cm^−1^ are attributed to the asymmetric and symmetric stretching of C-O-C bonds in the ester group, further confirming the presence of the ester carbonyl. All of this evidence confirms the successful synthesis of the target product HPEI-g-PDMAEMA.

### 2.3. Thermogravimetric Analysis (TGA)

**Figure 2:**
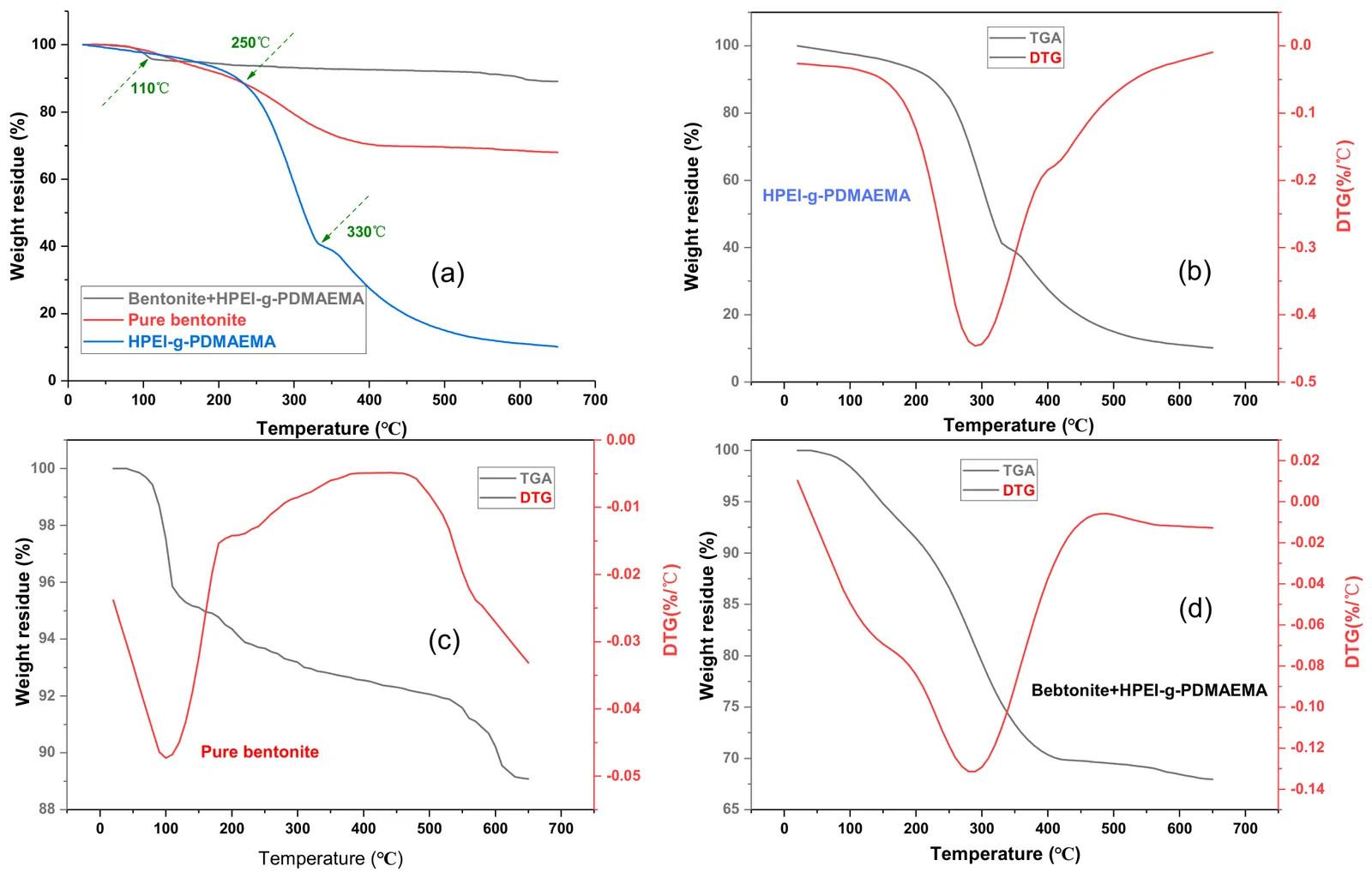
TGA curves of (**a**) overlay comparison of the three samples, (**b**) HPEI-g-PDMAEMA, (**c**) pure bentonite, and (**d**) bentonite treated with HPEI-g-PDMAEMA.

TGA was used to study the thermal stability of HPEI-g-PDMAEMA and its inhibitory effect on bentonite hydration (Figure 2a–d). As shown in the overlay comparison (Figure 2a), the weight loss from room temperature to 200 °C is mainly due to the evaporation of interlayer adsorbed water [41]. The weight loss of bentonite at ≥200 °C is attributed to the dehydroxylation of bentonite and the degradation of organic components [42] (Figure 2b). Compared with pure bentonite, the weight loss of bentonite treated with HPEI-g-PDMAEMA below 150 °C was significantly reduced (Figure 2d), indicating that the polymer effectively inhibited the formation of a hydrated film on the clay particle surface through electrostatic adsorption and hydrophobic interactions, reducing the amount of interlayer water adsorbed. Furthermore, HPEI-g-PDMAEMA itself showed small weight loss below 250 °C, demonstrating good thermal stability (Figure 2c). Around 250 °C, the weight loss curve of the composite lies between those of pure bentonite and pure polymer, further confirming the firm adsorption of the polymer onto the clay surface. These results indicate that HPEI-g-PDMAEMA effectively inhibits the hydration swelling of bentonite while possessing sufficient thermal stability to meet the requirements of high-temperature drilling fluid environments.

### 2.4. The Effect of pH on Zeta Potential

**Figure 3:**
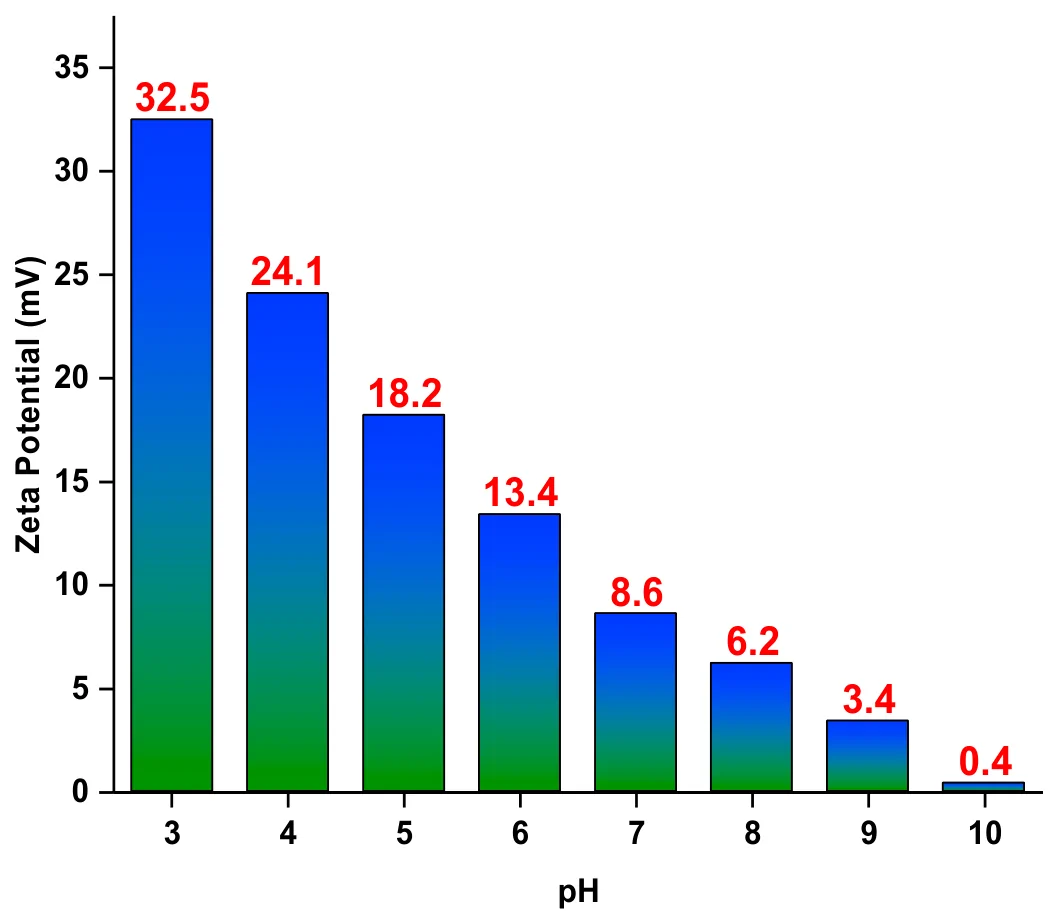
Zeta potential of HPEI-g-PDMAEMA aqueous solution versus pH variation.

The charged state of polymers in aqueous solutions directly affects their ability for electrostatic adsorption onto clay particles. Therefore, the zeta potential of the HPEI-g-PDMAEMA aqueous solution at different pH values was measured, as shown in Figure 3. The zeta potential decreased monotonically with increasing pH. Under strongly acidic conditions (pH = 3), the zeta potential was as high as +32.5 mV. This is because all amine groups on the polymer chain (including primary, secondary, and tertiary amines from HPEI and tertiary amine groups from DMAEMA segments) are highly protonated in acidic environments, forming a large number of ammonium ions, resulting in the highest positive charge density. As pH increased from 3 to 7, the zeta potential gradually decreased to +8.6 mV due to reduced protonation: some amine groups transitioned from a protonated to free base form, reducing the positive charge density. When pH further increased to 8–9, the zeta potentials were +5.12 mV (pH = 8) and +1.44 mV (pH = 9), respectively. Although these are significantly lower than those under acidic conditions, the polymer remained positively charged, which is crucial for enhancing clay adsorption and exerting shale inhibition. At pH = 10, the zeta potential approached zero (+0.07 mV), indicating that most amine groups were deprotonated and that the polymer was near its isoelectric point. This is consistent with the distinct pKa values of HPEI and PDMAEMA (approximately 8.5 and 7.5, respectively), and the grafting ratio influences the overall pH-zeta potential profile: higher PDMAEMA content shifts the isoelectric point to a slightly lower pH, whereas a higher HPEI content maintains a more positive surface charge over a broader pH range [43]. Notably, over the entire tested pH range (3–10), the zeta potential remained positive, without reversal from positive to negative, indicating that the polymer does not cause charge reversal on the clay surface under different pH conditions, favoring stable adsorption.

Thus, HPEI-g-PDMAEMA exhibits typical pH-responsive positive charge characteristics. In the weakly alkaline environment typically maintained in drilling fluids (pH = 8–9), the polymer retains a moderate positive charge, enabling effective adsorption onto the clay surface to exert inhibition while avoiding excessive flocculation caused by excessively high charge. This pH-tunable charge characteristic is an important foundation for the dual-responsive function of “electrostatic adsorption + thermosensitive enhancement”.

### 2.5. Effect of Polymer on Zeta Potential of Bentonite

**Figure 4:**
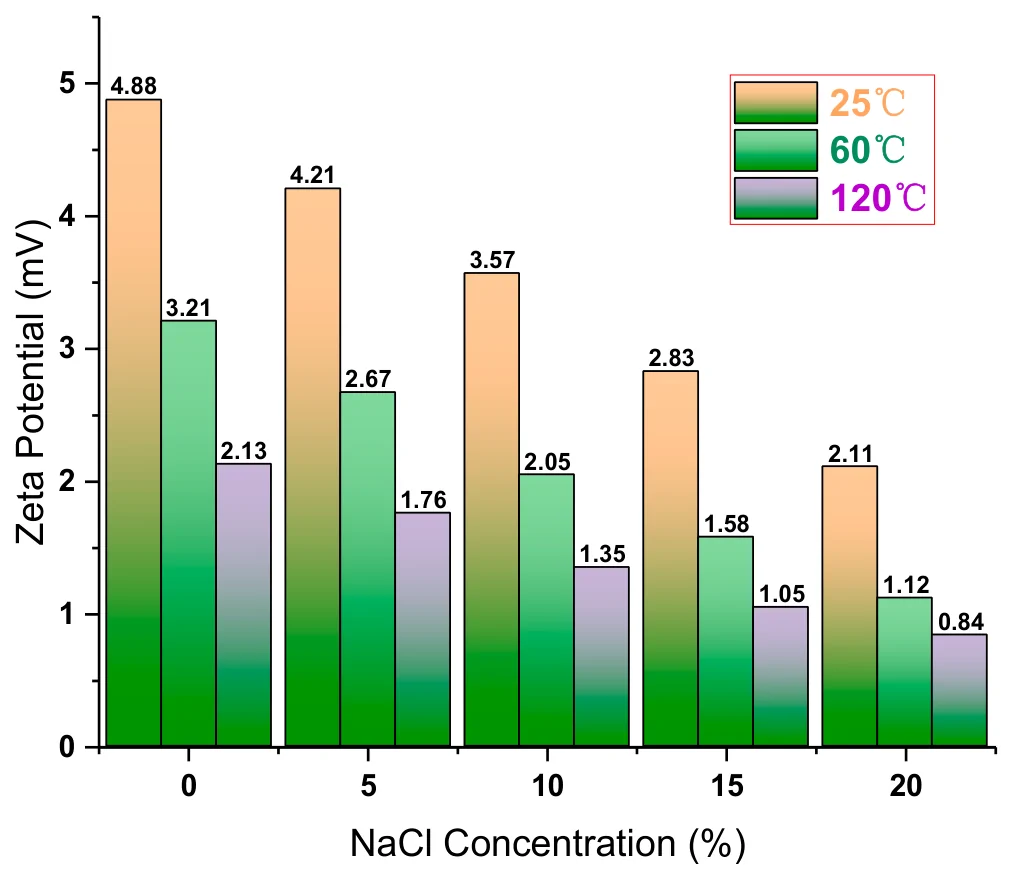
Zeta potential of bentonite after treatment with HPEI-g-PDMAEMA under different conditions.

To evaluate the adsorption stability of HPEI-g-PDMAEMA under different conditions, the zeta potential of bentonite treated with the polymer was measured at various temperatures and NaCl concentrations. The zeta potential of pure bentonite at 25 °C without salt was −28.5 mV, indicating a strong negative charge on its surface. After treatment with 1.5% HPEI-g-PDMAEMA, the zeta potential under the same conditions increased to +4.88 mV, confirming that the polymer successfully neutralized the negative charge on the clay surface via electrostatic adsorption. As shown in Figure 4, with increasing temperature, the zeta potential of the treated bentonite decreased: without salt, it decreased from +4.88 mV at 25 °C to +3.21 mV at 60 °C, and further to +2.13 mV at 120 °C. This is attributed to the increased deprotonation of the DMAEMA segments at high temperatures, reducing the positive charge of the polymer. As NaCl concentration increased, the zeta potential also gradually decreased, but under the extreme conditions of 120 °C and 20% NaCl, it remained at +0.84 mV, without charge reversal. This indicates that HPEI-g-PDMAEMA can still stably adsorb onto the clay surface even in high-temperature, high-salinity environments, maintaining effective electrostatic anchoring.

### 2.6. Thermosensitive Behavior

**Figure 5:**
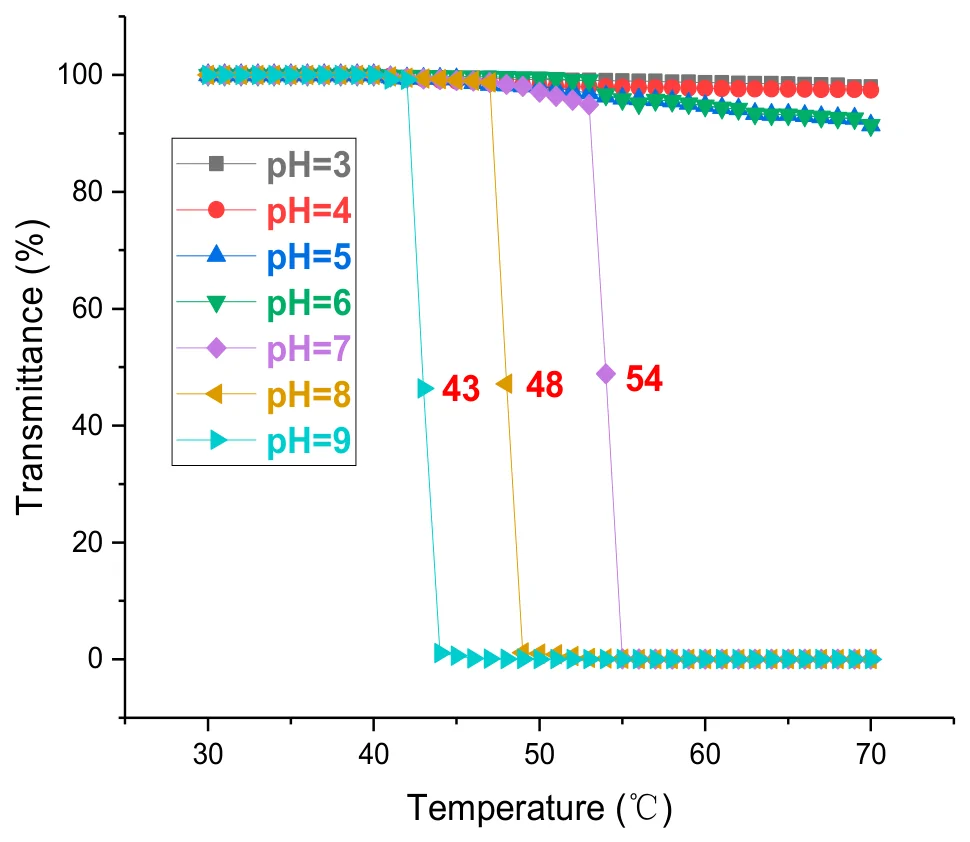
Temperature sensitivity trend of HPEI-g-PDMAEMA aqueous solution with pH variation.

To investigate the temperature response of the polymer, the transmittance of HPEI-g-PDMAEMA solutions at different pH values as a function of temperature was examined. As shown in Figure 5, the thermosensitive behavior was significantly affected by pH. At pH = 3, the solution remained transparent throughout the heating process, with no noticeable change in transmittance, indicating strong hydrophilicity and no phase transition under these conditions. Transitioning from acidic to neutral (pH = 7), the solution started to become turbid at about 50 °C, with an LCST of 54 °C. When the pH increased to weakly alkaline, the LCST decreased to 43 °C, indicating the enhanced hydrophobicity of the polymer under alkaline conditions, with some segments already in a pre-aggregated state at room temperature. Drilling fluid systems are typically maintained at a weakly alkaline pH of 8–9. Within this pH range, the LCST of HPEI-g-PDMAEMA is 43–48 °C, which exactly covers the typical temperature increase experienced by drilling fluid from surface circulation to the bottomhole. This indicates that the inhibitor has good solubility at surface temperature and can effectively undergo a hydrophobic transition upon entering the bottomhole, thereby enhancing inhibition performance. At pH = 3, the absence of a phase transition is attributed to the strong protonation of the tertiary amine groups in PDMAEMA, forming hydrophilic quaternary ammonium species that remain fully hydrated even at elevated temperatures. This is consistent with the high positive zeta potential (+32.5 mV, Figure 5) present under strongly acidic conditions.

**Figure 6:**
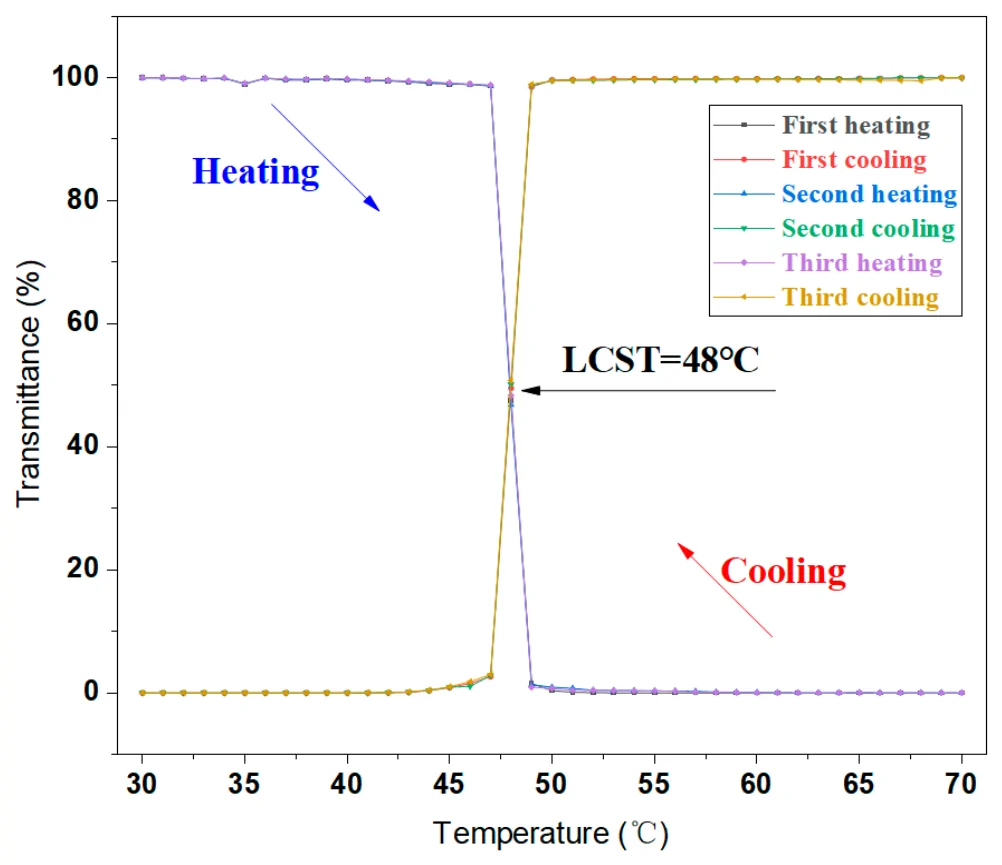
The light transmittance curves of HPEI-g-PDMAEMA in pure water during three consecutive heating–cooling cycles.

Figure 6 presents the transmittance curves of HPEI-g-PDMAEMA during three consecutive heating–cooling cycles, demonstrating fully reversible phase transition behavior. In each cycle, the transmittance dropped sharply above the LCST and recovered to above 98% upon cooling, with only slight attenuation over repeated cycles. This excellent reversibility is attributed to the cooperative dehydration and rehydration of PDMAEMA side chains, which allows the polymer chains to collapse and redisperse without significant aggregation or degradation. These results confirm that the temperature-responsive behavior of HPEI-g-PDMAEMA is fully reversible, which is essential for practical drilling applications in which the inhibitor experiences repeated temperature fluctuations during circulation.

### 2.7. Particle Size Test

To confirm the thermosensitive aggregation behavior of the polymer, the hydrodynamic diameter of the polymer aqueous solution (0.5 wt%) at different pH values was measured using a nanoparticle size analyzer (Table 5). At 30 °C, the initial particle size slightly increased with increasing pH (24→35 nm), indicating slight chain collapse due to deprotonation. When the temperature was raised above the LCST, the particle sizes of all three samples sharply increased (200–355 nm), and the temperature at which the particle size jump occurred was consistent with the LCST determined by transmittance measurements. Previous studies have shown that thermosensitive polymer aggregation on the clay surface can further induce bridging between clay particles, forming micron-sized aggregates and thereby achieving physical plugging of shale microfractures [40].

To verify reversibility at the colloidal level, we also monitored the hydrodynamic diameter during one heating–cooling cycle at pH 8. Upon heating from 30 °C to 55 °C, the diameter increased from 29.4 nm to 274.5 nm; upon cooling back to 30 °C, it returned to 32.1 nm (within 10% of the initial value). This reversible size change, together with the transmittance cycling data (Table 6), confirms that the temperature-induced aggregation is a reversible hydrophobic collapse rather than irreversible precipitation.

### 2.8. Shale Inhibition Performance Evaluation

To test whether the pH- and temperature-responsive properties translate into practical clay hydration control, we evaluated the polymer’s performance under simulated high-temperature and high-salinity conditions. All these inhibition tests were carried out at a controlled pH of 8.0 ± 0.1 to mimic the actual drilling fluid environment and to ensure the LCST trigger remained consistent.

#### 2.8.1. Hot-Rolling Recovery Rate Test

**Figure 7:**
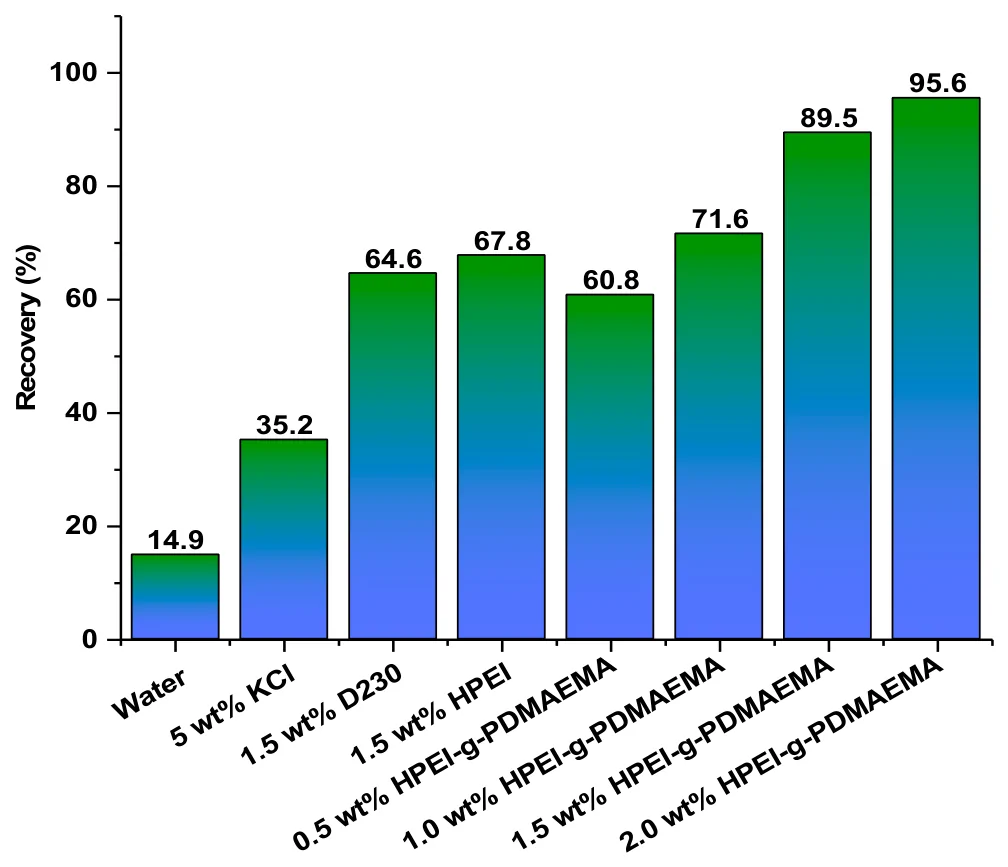
Recovery rates of shale fragments in different inhibitor solutions (aging at 150 °C for 16 h, pure water).

Figure 7 presents the hot-rolling recovery rates of shale cuttings treated with different inhibition systems as a function of temperature. A 5 wt% KCl solution was used as a conventional reference, while the two polymers (HPEI and HPEI-g-PDMAEMA) were evaluated at a low concentration of 1.5 wt%. At 25 °C, both polymers outperformed KCl: the recoveries for HPEI and HPEI-g-PDMAEMA were 92.5% and 94.1%, respectively, compared to 82.5% for KCl. As the temperature increased, the recovery of HPEI continuously decreased to 81.6% at 180 °C, indicating a loss of inhibition capacity under thermal stress. In striking contrast, HPEI-g-PDMAEMA exhibited a non-monotonic temperature dependence: its recovery first increased to a maximum of 94.1% at 150 °C and then slightly decreased to 91.9% at 180 °C. This increase exactly coincides with the LCST range of the polymer (≈45–48 °C at pH = 8). Below the LCST, the polymer remains hydrophilic and exhibits moderate adsorption; upon heating above the LCST, the PDMAEMA segments undergo hydrophobic collapse, which enhances surface coverage and water-blocking efficiency, thereby maximizing inhibition near 150 °C. Even at 180 °C, the recovery remains above 90%, demonstrating that the thermo-induced hydrophobic association is largely retained under harsh thermal conditions. These results confirm that the dual-responsive design not only surpasses conventional KCl at all tested temperatures but also provides an “on-demand” enhancement that is specifically triggered in the high-temperature region.

#### 2.8.2. Salt Resistance Evaluation

The hot-rolling temperature was fixed at 150 °C with an inhibitor dosage of 1.5 wt%. The effect of salt type and concentration on shale recovery is shown in Table 7. For NaCl, as concentration increased from 0 to 20 wt%, HPEI recovery dropped from 85.3% to 32.5%, while HPEI-g-PDMAEMA recovery decreased from 94.1% to 68.4%. For divalent salts, the recoveries were consistently lower: at 20 wt% CaCl_2_, HPEI and HPEI-g-PDMAEMA achieved 28.1% and 60.3% recovery, respectively; in MgCl_2_, the corresponding values were 24.3% and 57.6%. Notably, Mg^2+^ exhibited a slightly stronger inhibitory effect on recovery than Ca^2+^ at each concentration, likely due to its smaller hydrated radius and higher charge density, which enabled more effective compression of the clay electrical double layer. The graft copolymer significantly outperformed unmodified HPEI across all salt types and concentrations, maintaining recoveries above 77% at 10 wt% for both divalent salts, whereas HPEI recovery fell below 55% under the same conditions.

The superior salt resistance of HPEI-g-PDMAEMA is attributed to its dual anchoring mechanism. The protonated amine groups on the hyperbranched backbone provide strong electrostatic anchoring onto clay surfaces, while the thermo-responsive PDMAEMA segments, when collapsed above LCST, form a hydrophobic gel layer that is largely insensitive to ionic strength. Although multivalent cations partially screen electrostatic interactions via charge shielding and competitive adsorption, the hydrophobic association of DMAEMA segments remains operative, serving as a robust backup anchoring force that preserves inhibition performance even under high-salinity conditions. Additionally, the multiple amine groups of the hyperbranched architecture provide additional adsorption sites, compensating for the partial loss of electrostatic binding in saline environments. This combination makes the graft copolymer suitable for high-temperature, high-salinity drilling applications.

#### 2.8.3. Bentonite Linear Swelling Experiment

**Figure 8:**
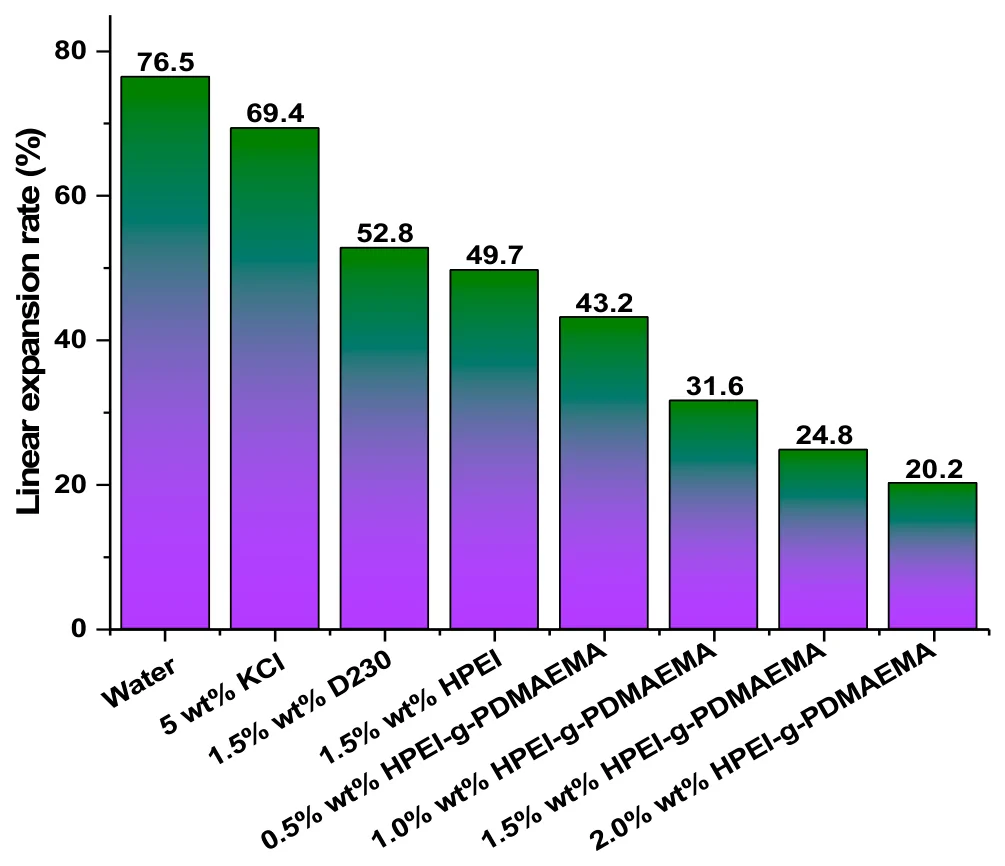
Linear expansion rate of different inhibitors.

To investigate the effect of temperature on the ability of the inhibitor to inhibit bentonite hydration swelling, the 8 h linear swelling height of different systems was measured at 25–60 °C with a fixed inhibitor dosage of 1.5 wt%. As shown in Figure 8, the linear swelling height of the blank water group slightly increased with temperature (12.5→13.8 mm), indicating that high temperature exacerbates clay hydration. The linear swelling height of HPEI did not change significantly with temperature (4.5–4.8 mm), showing no thermosensitivity. In contrast, HPEI-g-PDMAEMA exhibited significant temperature-responsive behavior: at 25 °C, the linear swelling height was 4.3 mm, which is similar to that of HPEI; at 40 °C, it decreased to 3.5 mm; at 50 °C, it sharply dropped to 2.4 mm; and at 60 °C, it further decreased to 2.1 mm. The inhibition performance (relative to water) increased from 65.6% at 25 °C to 84.1% at 60 °C. This result is highly consistent with the LCST measured in Section 2.6 (48 °C at pH = 8), confirming that when the temperature exceeds the LCST, the DMAEMA segments undergo hydrophobic aggregation, forming a dense hydrophobic film on the clay surface, thereby effectively inhibiting the interlayer hydration swelling of bentonite. These results further support the thermo-sensitive enhancement mechanism.

#### 2.8.4. Bentonite Slurry-Making Experiment

**Figure 9:**
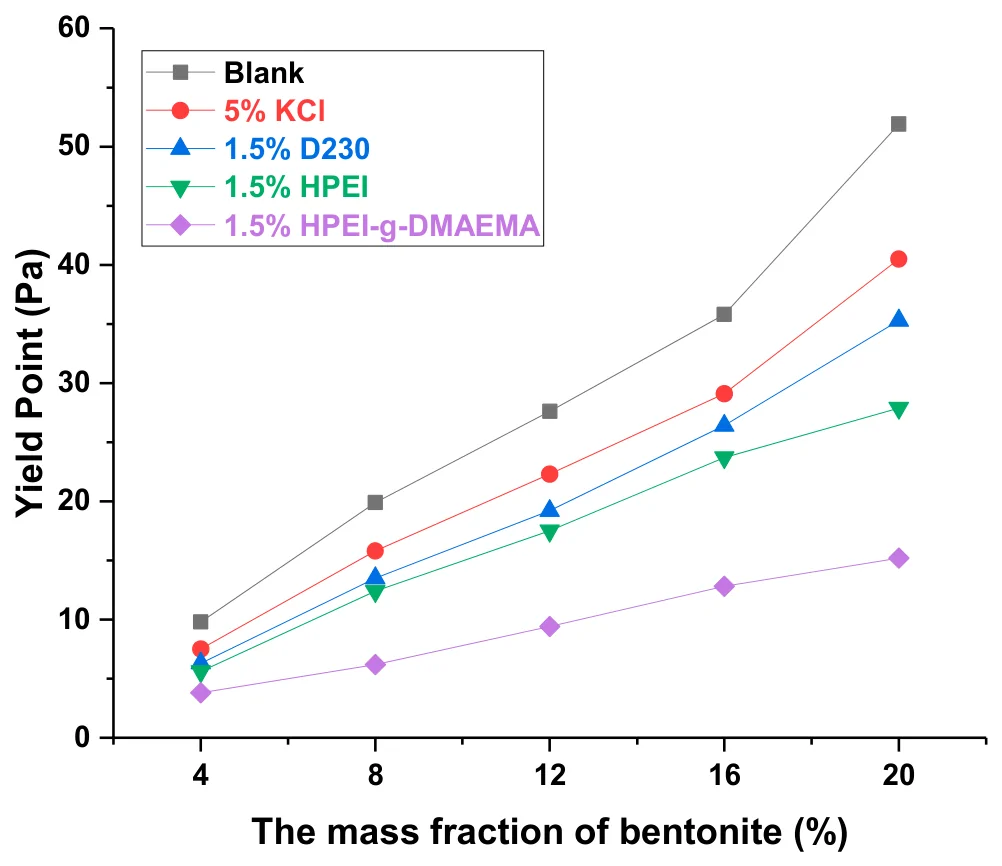
Yield point values of bentonite slurries with different inhibitor systems.

The bentonite slurry-making experiment effectively verifies the inhibition effect of shale inhibitors on clay hydration and dispersion. As shown in Figure 9, with increasing bentonite content, the yield point of all systems gradually increased. HPEI-g-PDMAEMA consistently exhibited the lowest YP across all concentrations, followed by HPEI, D230, KCl, and the blank. At a 20% bentonite content, the YP values were 51.9 Pa (blank), 44.5 Pa (5 wt% KCl), 38.5 Pa (1.5 wt% D230), 27.9 Pa (HPEI), and 15.2 Pa (HPEI-g-PDMAEMA). To provide a more comprehensive rheological characterization, we further measured the full Bingham parameters at a 20 wt% bentonite content for all five systems at both 25 °C and 60 °C, as summarized in Table 8. The results show that HPEI-g-PDMAEMA not only reduces YP but also significantly decreases AV, PV, gel strengths, and the YP/PV ratio compared to KCl, D230, and unmodified HPEI. This enhanced rheological performance is attributed to the dual action of the graft copolymer: (1) the protonated amine groups on the hyperbranched HPEI backbone neutralize the negative charge on the clay surface via electrostatic adsorption, weakening electrostatic repulsion and preventing extensive particle delamination; (2) the long PDMAEMA side chains provide steric hindrance that prevents particle bridging and aggregation. Unmodified HPEI lacks the steric hindrance effect, while KCl and D230 rely primarily on charge screening or hydrogen bonding, which are less effective than the combined electrostatic + steric mechanism of the graft copolymer.

#### 2.8.5. API Filtration Performance

The API filtrate volume and filter cake thickness of bentonite slurries treated with different inhibitors are presented in Table 9. The blank slurry exhibited a filtrate volume of 26.8 mL and a cake thickness of 3.2 mm, indicating a loose and highly permeable filter cake. Upon the addition of 1.5 wt% unmodified HPEI, the filtrate volume decreased to 18.5 mL with a corresponding cake thickness of 2.5 mm, attributable to the electrostatic neutralization of clay particles, which promotes flocculation and denser particle packing. Notably, HPEI-g-PDMAEMA further reduced the filtrate volume to 12.3 mL and the cake thickness to 1.8 mm, representing reductions of 54% and 44%, respectively.

The superior performance of the graft copolymer is attributed to two synergistic effects. First, the hyperbranched HPEI backbone provides abundant cationic anchoring sites that effectively neutralize clay surface charges, facilitating the formation of a compact cake with lower permeability. Second, the flexible PDMAEMA side chains act as polymer bridges between adjacent clay particles, reinforcing the structural integrity of the cake and reducing its susceptibility to fluid erosion. The simultaneous reduction in both filtrate volume and cake thickness confirms that HPEI-g-PDMAEMA not only inhibits clay hydration but also constructs a tighter physical barrier against fluid invasion.

### 2.9. Mechanism Analysis

**Figure 10:**
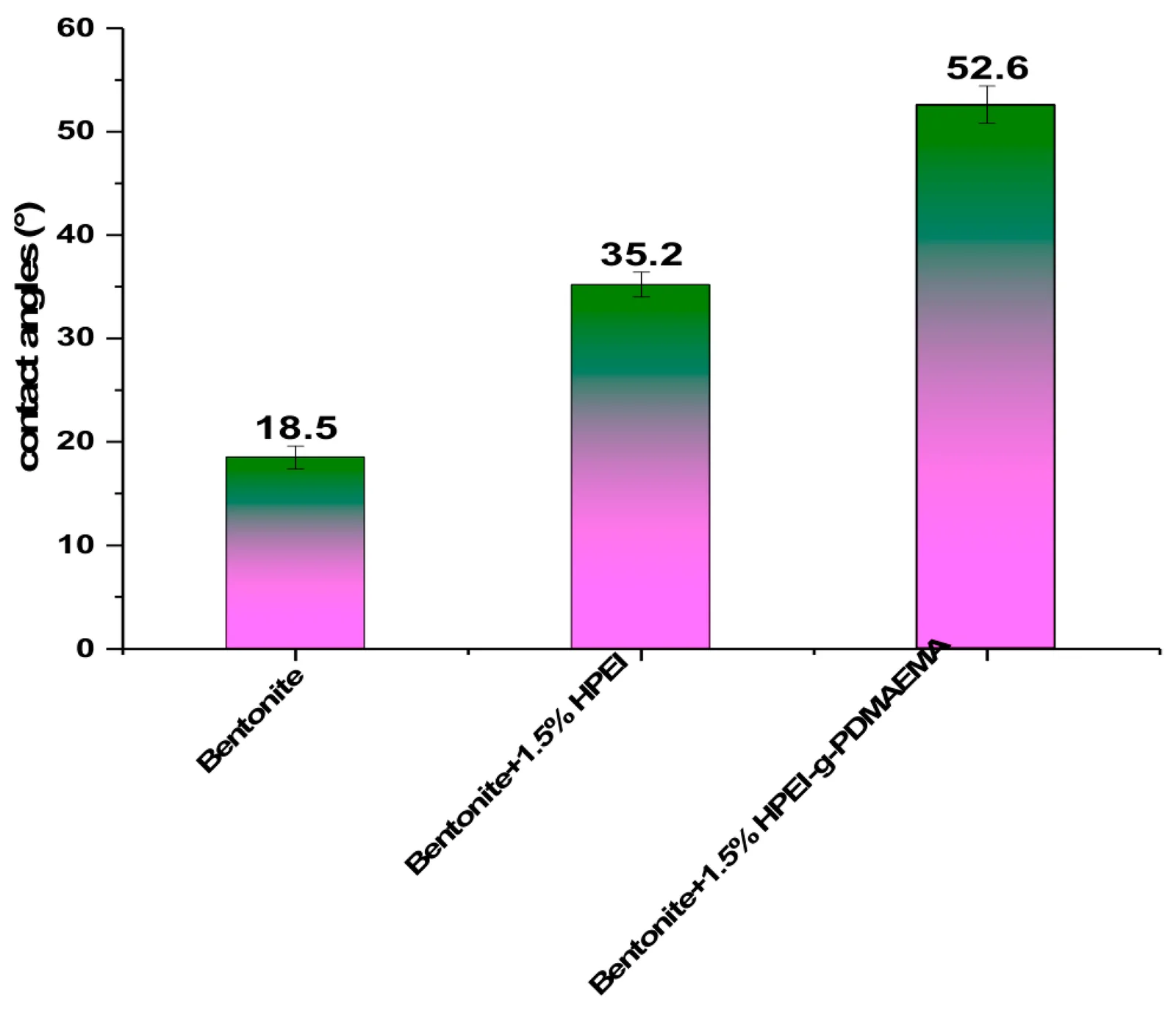
Water contact angles on a clay pellet.

As shown in Figure 10, the water contact angle of untreated bentonite was only 18.5°, indicating strong hydrophilicity. After treatment with HPEI, the contact angle increased to 35.2°, suggesting partial hydrophobization. Notably, HPEI-g-PDMAEMA treatment further increased the contact angle to 52.6°, confirming that the graft copolymer forms a more effective hydrophobic barrier on the clay surface, preventing water from entering further into the bentonite particle crystals [44]. This enhanced hydrophobicity is attributed to the hydrophobic aggregation of DMAEMA segments, which blocks water molecules from accessing the clay surface and thus inhibits hydration swelling.

**Figure 11:**
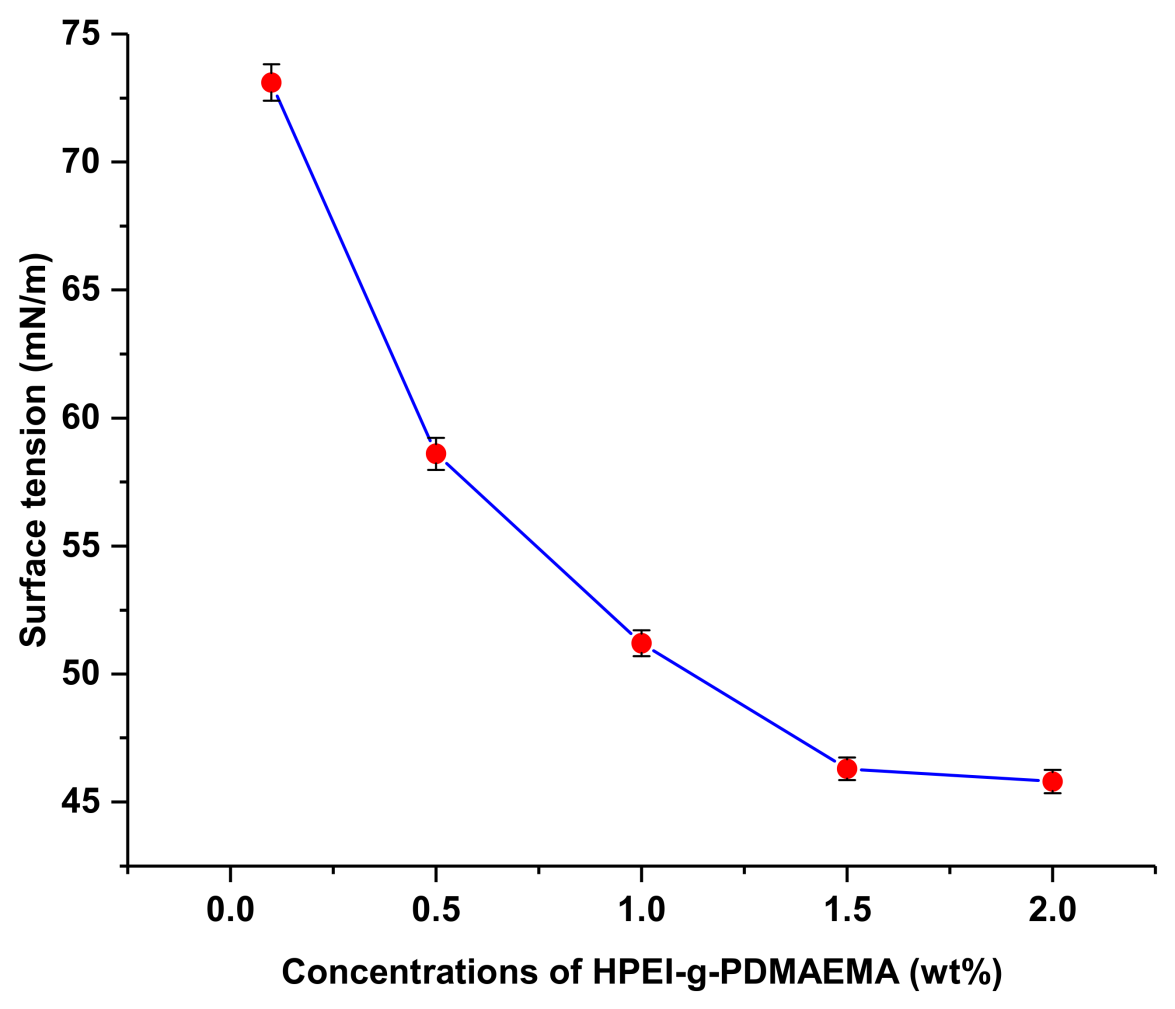
Surface tension of different concentrations of HPEI-g-PDMAEMA solutions.

Figure 11 shows that with the increase in HPEI-g-PDMAEMA concentration, the surface tension of the solution gradually decreased. When the concentration reached 1.5 wt%, the surface tension decreased from 73.1 mN/m to 46.3 mN/m, and further increasing the concentration to 2.0 wt% caused no significant change (45.8 mN/m), indicating a critical micelle concentration (CMC) of approximately 1.5 wt%. The reduction in surface tension weakens the capillary force that drives the drilling fluid filtrate into shale microfractures [45], thereby contributing to the inhibition performance..

**Figure 12:**
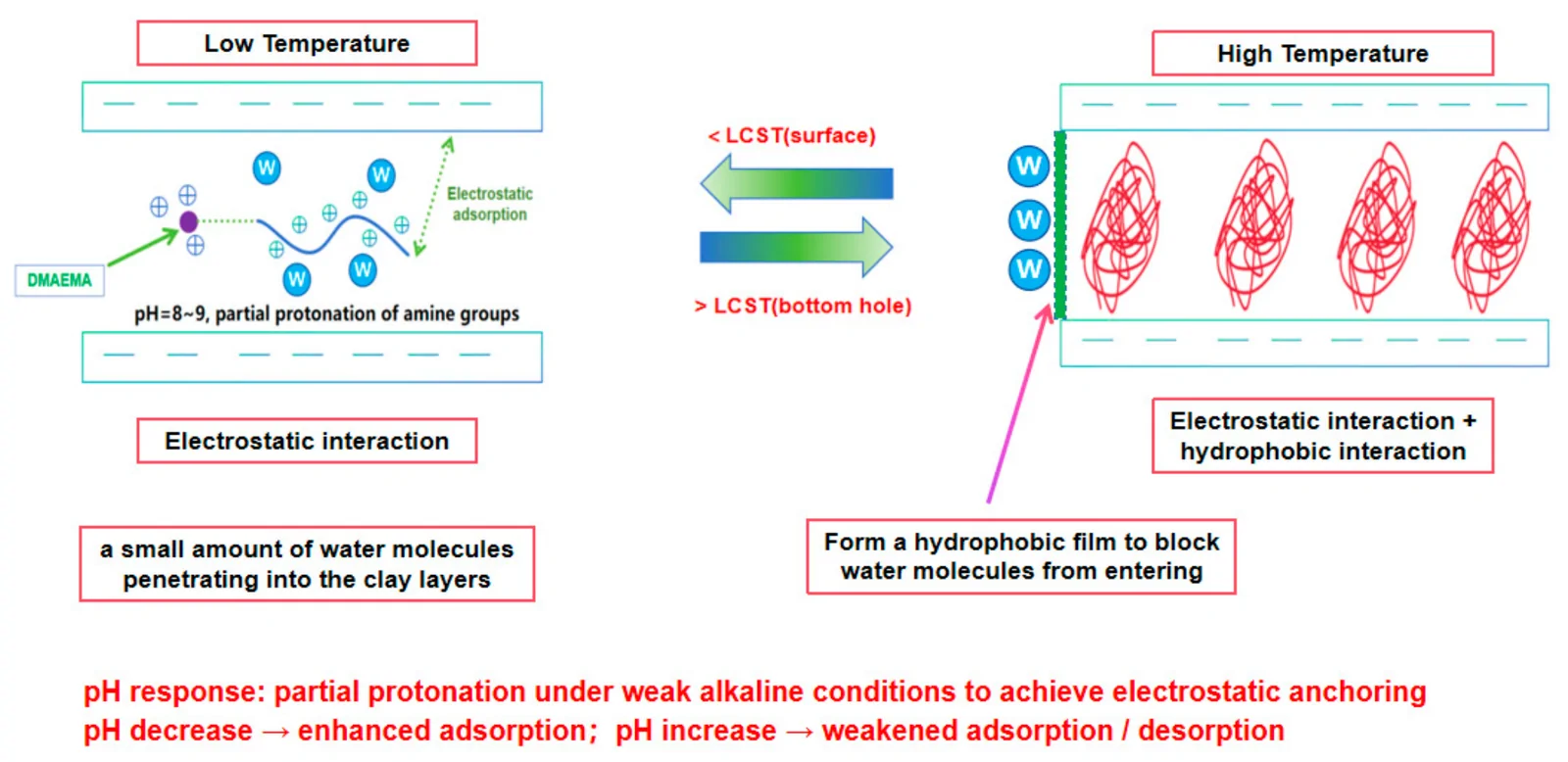
Schematic diagram of the pH/temperature dual-responsive inhibition mechanism of HPEI-g-PDMAEMA.

From the experimental results, the mechanism of action of the polymer can be deduced, as illustrated in Figure 12. The analysis is as follows:

In terms of electrostatic adsorption and pH responsiveness, the amine groups on the HPEI backbone of the polymer are partially protonated in the weakly alkaline drilling fluid environment, acquiring positive charges. These adsorb onto the negatively charged clay particle surface via electrostatic attraction, neutralizing the negative charge and reducing electrostatic repulsion between particles. At the same time, the pH-responsive characteristics of the HPEI backbone further modulate the adsorption strength: as shown in Figure 5, at pH = 8–9 the polymer retains a moderate positive charge, avoiding excessive flocculation; when the pH decreases to acidic, the protonation of amine groups is enhanced, and the tertiary amine groups in DMAEMA also become protonated, leading to stronger adsorption; when the pH increases above 10, beyond the pKa of HPEI and DMAEMA, deprotonation occurs, the polymer approaches its isoelectric point, and adsorption capacity weakens—achieving “on-demand anchoring” and potential reversible desorption.

In terms of temperature responsiveness, when the temperature rises above 45 °C, the DMAEMA segments undergo a hydrophilic-to-hydrophobic transition, leading to two effects: (1) the hydrophobic effect promotes increased adsorption of the polymer onto the clay surface; (2) the adsorbed polymer chains form a denser hydrophobic film on the clay surface, blocking water molecules from entering. The synergy of these two effects allows the inhibitor to maintain good dispersion during low-temperature surface circulation and automatically enhance inhibition upon entering the high-temperature bottomhole, achieving “on-demand inhibition” adaptive regulation. That is, pH responsiveness relies on HPEI (high pKa, still protonated under alkaline conditions), and temperature responsiveness relies on PDMAEMA (low pKa, neutral under alkaline conditions, hydrophobic collapse upon heating), constituting a dual response.

In terms of the gel science perspective, the untreated bentonite suspension forms a highly hydrated, negatively charged physical gel. Upon the addition of HPEI-g-PDMAEMA, the polymer electrostatically anchors onto clay platelets, partially neutralizes the surface charge, and reduces the gel’s water affinity. When the temperature exceeds the LCST, the PDMAEMA segments collapse, creating hydrophobic patches on clay surfaces. This leads to a transition from a hydrophilic clay gel to a hydrophobically associated gel with dramatically lower water uptake and higher resistance to salt and heat. Such a gel-level understanding explains why the inhibitor performs better at high temperatures than conventional inhibitors.

For a comparison to the HPEI-PNIPAM-PO terpolymer recently reported by Hu et al. [40], see Table 10. In Hu et al.’s literature system, PNIPAM is used as the thermosensitive segment, with an LCST of about 32 °C, which is close to or below the surface temperature, meaning that the inhibitor may prematurely collapse before reaching the target zone, reducing the effective concentration. Moreover, PNIPAM mainly interacts with the clay surface via hydrogen bonds through amide groups, with low binding energy, making it prone to desorption at high temperatures and under high-salinity conditions. In contrast, in this work, DMAEMA has an LCST of about 45–48 °C in alkaline drilling fluid, which is significantly above the surface temperature, ensuring good dispersion at the surface and only triggering hydrophobic collapse in the high-temperature bottomhole region, achieving “temperature-targeted” on-demand inhibition. Furthermore, the HPEI backbone remains partially protonated under weakly alkaline conditions, providing strong electrostatic anchoring onto the clay surface, with anchoring strength much higher than that of hydrogen bonding. This work adopts a simpler binary molecular design (without introducing a hydrophobic monomer such as PO), offering advantages in synthesis simplicity and water solubility.

## 3. Conclusions

(1) A pH/temperature dual-responsive graft copolymer, HPEI-g-PDMAEMA, was successfully synthesized and characterized. The optimal synthesis conditions (HPEI/DMAEMA mass ratio 1:2, 2.4% AIBN, 70 °C, 10 h) yielded a grafting ratio of 35.2% and a relative molecular weight of 84,298 g/mol (PEO/PEG standards).
(2) The polymer exhibits a pH-tunable LCST, decreasing from 54 °C at pH 7 to 43 °C at pH 9. In the weakly alkaline range (pH 8–9) typical of drilling environments, the LCST (≈45–48 °C) lies well above surface temperatures but below common downhole temperatures. This temperature-targeted trigger ensures that the polymer remains well-dispersed during surface circulation and undergoes reversible hydrophobic collapse only upon entering the hot bottomhole region, as confirmed by transmittance cycling and DLS measurements.
(3) Mechanistically, the HPEI backbone remains partially protonated at pH 8–9, as confirmed by a positive zeta potential (+5 mV at pH 8), enabling strong electrostatic anchoring onto negatively charged clay surfaces. Above the LCST, PDMAEMA segments undergo hydrophobic aggregation, as evidenced by a sharp increase in the hydrodynamic diameter (from ≈30 nm to >200 nm). This dual action transforms the hydrated clay into a hydrophobically associated gel with dramatically reduced water uptake. Consequently, the copolymer achieves a hot-rolling recovery of 94.1% at 150 °C (1.5 wt% dosage), significantly outperforming KCl, D230, and unmodified HPEI, and maintains recovery above 60% even in 20 wt% divalent brines. The treated clay surface shows a water contact angle of 52.6° compared to 18.5° for untreated clay.
(4) This work provides a molecular-level design strategy for stimuli-responsive polymer–clay interfaces, where pH and temperature serve as orthogonal triggers to reversibly switch surface wettability and hydration resistance. The binary HPEI-g-PDMAEMA system offers a simpler and more robust alternative to ternary copolymers, with a well-matched LCST window and strong electrostatic anchoring.

Limitations and Future Work:

This study provides indirect evidence for the proposed multi-scale stabilization mechanisms of HPEI-g-PDMAEMA through macroscopic and colloidal characterization. However, several limitations remain: (i) the grafting ratio and copolymer structure are not directly determined, owing to current time and instrument constraints that preclude high-field ^1^H NMR analysis; (ii) purification is restricted to laboratory-scale dialysis; (iii) the environmental fate and ecotoxicity are unassessed; and (iv) direct visualization of the temperature-triggered interfacial switching is lacking.

To address these gaps, future work will focus on the following:

1. Environmental assessment: Future work should perform standardized biodegradation tests and multi-species ecotoxicity assays (e.g., algae, daphnids) to establish a comprehensive risk profile.
2. Scalable purification and pilot validation: It should develop membrane ultrafiltration or continuous diafiltration to replace dialysis, followed by pilot-scale synthesis and field trials to confirm economic feasibility and long-term stability.
3. Direct mechanistic visualization: Researchers should employ variable-temperature AFM, QCM-D, cryo-SEM, or CLSM to directly observe the interfacial film, 3D network, and filter-cake microstructure, providing visual evidence for the self-reinforcement mechanism.
4. Structural characterization and performance optimization: Studies should be focused on determining the grafting ratio using alternative techniques (elemental analysis, TGA, or XPS) for now, and revisiting precise 1H NMR confirmation when accessible. Based on structure-property insights, further architectural tuning should be pursued to enhance salt tolerance and thermal stability for ultra-deep well applications (>200 °C).

## 4. Materials and Methods

### 4.1. Materials

The materials used were as follows: HPEI (M_w_ = 60,000, industrial grade, Macklin Biochemical Technology Co., Ltd., Shanghai, China.); DMAEMA (analytical grade, Aladdin Reagent Co., Ltd (Shanghai, China)., purified by vacuum distillation to remove inhibitor); AIBN (analytical grade, recrystallized from ethanol); sodium bentonite (industrial grade, Zhejiang FengHong New Material Co., Ltd., Huzhou, Zhejiang); shale cuttings (taken from the Longmaxi Formation in Changning area, Sichuan, crushed and sieved through a 6-mesh screen); polyether amine (D230) (industrial grade, Shandong Rongwei Chemical Co., Ltd., Jinan, Shandong); NaOH, HCl, NaCl, CaCl_2_, MgCl_2_, etc. (all analytical grade, Shanghai Macklin Biochemical Technology Co., Ltd., Shanghai, China); distilled water prepared in the laboratory.

The instruments used were as follows: Fourier transform infrared spectrometer (Nicolet iS10, Thermo Fisher, Shanghai, China); gel permeation chromatograph (Waters 1515, Waters, Shanghai, China); thermogravimetric analyzer (TA Q500, TA Instruments, Shanghai, China); high-temperature hot-rolling oven (XGRL-4A, Qingdao Senxin Mechanical and Electrical Equipment Co., Ltd., Qingdao, Shandong); contact angle goniometer (Dongguan Jingyu Instrument Equipment Technology Co., Ltd., Dongguan, Guangdong); six-speed viscometer (ZNN-D6, Shandong Meike Instrument Co., Ltd., Qingdao, Shandong); nanoparticle size analyzer (Litesizer 500, Anton Paar, Shanghai, China). The mineralogical and clay mineral composition of the shale cuttings is shown in Table 11.

### 4.2. Preparation of Polymer

**Figure 13:**
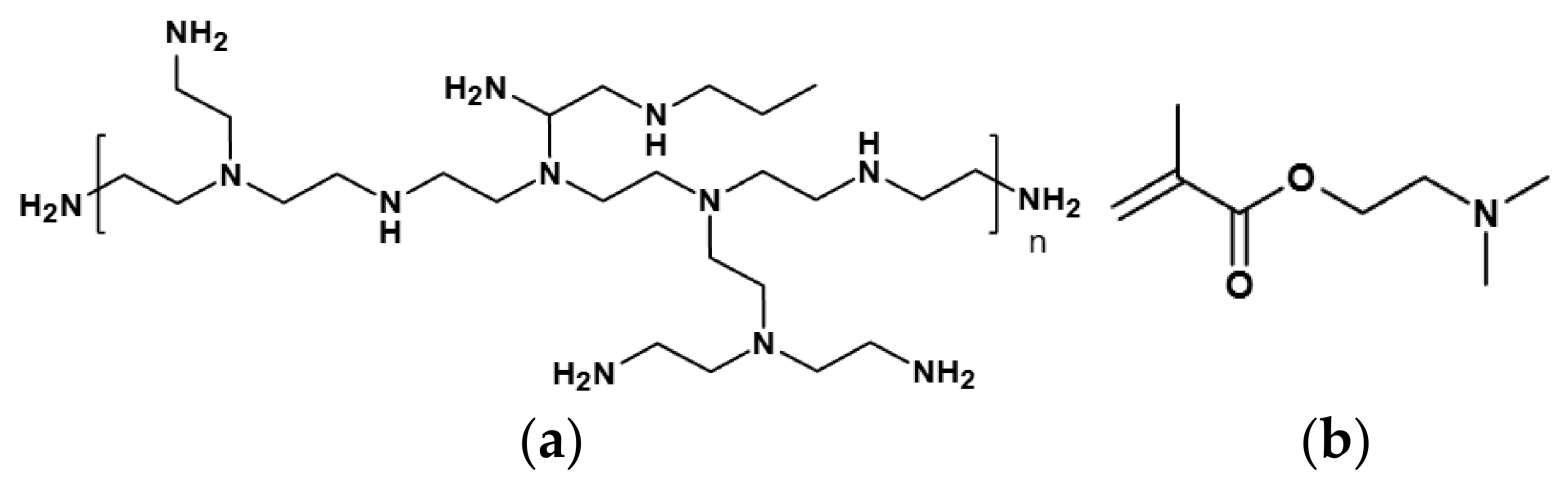
(**a**). Molecular structural formula of HPEI. (**b**). Molecular structural formula of DMAEMA.

**Figure 14:**
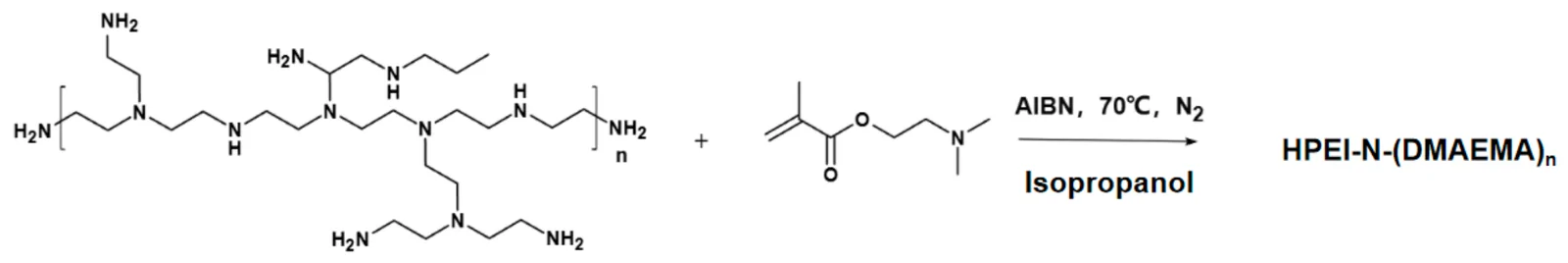
Schematic diagram of HPEI-g-PDMAEMA synthesis.

An amount of 5.0 g HPEI was dissolved in 50 mL of isopropanol, and N_2_ was bubbled for 30 min to remove oxygen. Specific amounts of DMAEMA monomer and AIBN were added, and the reaction was carried out under N_2_ protection at a set temperature for a specified time. After the reaction, the product was dialyzed against deionized water for 48 h (MWCO 8000), with the dialysate changed every 8 h to remove unreacted monomers and possible homopolymers, and finally freeze-dried to obtain the graft copolymer, denoted as HPEI-g-PDMAEMA. The molecular structures of HPEI and DMAEMA are shown in Figure 13a,b, respectively, and the synthesis process is shown in Figure 14. The grafting ratio was calculated according to Equation (1):

(1) $$\mathrm{Grafting}\;\mathrm{Ratio}=\frac{\left({W_{2}\;-\;W}_{1}\right)}{W_{1}}\times 100\%$$

where W_1_ is the mass of HPEI before grafting (g), and W_2_ is the mass of the product after grafting (g). The gravimetric value obtained from Equation (1) is used in this work as a practical measure for comparing samples synthesized under different conditions, rather than as an absolute structural parameter such as grafting density (number of side chains per backbone).

### 4.3. Polymer Characterization and Testing

#### 4.3.1. Fourier Transform Infrared (FTIR) Analysis

FTIR spectra were recorded on a PerkinElmer spectrometer at 25 °C. Samples were prepared by grinding with potassium chloride (KBr) to form pellets. Spectra were acquired across the wavenumber range of 400 cm^−1^–4000 cm^−1^.

#### 4.3.2. Thermogravimetric Analysis (TGA)

Using the Using TA Q500, thermogravimetric analyzer (TA Instruments, Shanghai, China), the temperature was increased from 20 °C to 650 °C at a heating rate of 10 °C/min and then stopped. Nitrogen was used as a protective gas during heating.

#### 4.3.3. Molecular Weight Measurement

Using the Agilent 1260 Infinity HT-GPC system, a polymer concentration of 2 mg/mL was used, and the eluent was 0.5 M NaNO_3_/20 mM phosphate buffer (pH = 7.0). The GPC system was calibrated using narrow-distribution polyethylene oxide/polyethylene glycol (PEO/PEG) standards. However, it should be noted that HPEI-g-PDMAEMA possesses a hyperbranched graft architecture significantly different with hydrodynamic behavior than linear standards. The reported M_w_ values are therefore relative rather than absolute. For this reason, we primarily use molecular weight data for comparative purposes among the samples synthesized under different conditions, rather than as absolute structural parameters.

#### 4.3.4. Temperature-Responsive Behavior Test

Then, 1.0 wt% polymer aqueous solutions were prepared; the pH was adjusted to different values with 0.1 mol/L HCl or NaOH. The change in light transmittance with temperature was measured using an Anton Paar nanoparticle size analyzer. The conditions were as follows: heating rate, 1.0 °C/min; termination temperature, 70 °C. The temperature at which transmittance dropped to 50% of the initial value was defined as the LCST. For the cycling test, the solution was alternately heated from 25 °C to 70 °C and cooled back down to 25 °C at a rate of 1.0 °C/min, and transmittance was recorded continuously over three cycles.

#### 4.3.5. Particle Size Measurement

Next, 1.0 wt% polymer aqueous solutions were prepared, and the hydrodynamic diameter of the polymer at different temperatures was measured using a dynamic light scattering instrument (Malvern Zetasizer Nano ZS90, Malvern, United Kingdom). Samples were equilibrated at the set temperature for 5 min before measurement; each sample was measured three times in parallel.

#### 4.3.6. Zeta Potential Measurement

Additionally, 0.1 wt% polymer aqueous solutions were prepared, the pH was adjusted to different values, and the zeta potential was measured at 25 °C. Each sample was measured three times and averaged.

### 4.4. Performance Evaluation of HPEI-g-PDMAEMA

#### 4.4.1. Hot-Rolling Recovery Rate Test

An amount of 50 g of shale cuttings was added to 350 mL of polymer solution (inhibitor dosage 1.5 wt%), placed in an aging cell, which was rotated end-over-end at 30 rpm, hot-rolled at a set temperature for 16 h, cooled, and then passed through a 40-mesh sieve. The cuttings retained on the sieve were dried at 105 °C to constant weight, and the hot-rolling recovery rate was calculated. Each experiment was repeated three times.

#### 4.4.2. Bentonite Linear Swelling Test

An amount of 1.0 g sodium bentonite was pressed at 10 MPa for 10 min to form a tablet of 25 mm diameter and placed in a linear swelling meter, and then a polymer solution was added. The light swelling height was recorded for 8 h. Each experiment was repeated three times.

#### 4.4.3. Bentonite Slurry Making Experiment

A specific amount of sodium bentonite was added to distilled water to prepare bentonite slurries with different mass fractions (4%, 8%, 12%, 16%, 20%). Then, 1.5 wt% inhibitor was added; the solution was stirred at 10,000 rpm for 20 min, and the yield point was measured using a six-speed rotational viscometer at 600 and 300 rpm. The yield point was calculated as the difference between the 600 rpm and 300 rpm readings (Bingham plastic model). The yield point measured in this experiment reflects the gel strength of the bentonite suspension, which is a key parameter for clay gel systems.

#### 4.4.4. Salt Resistance Evaluation

Different concentrations of NaCl, CaCl_2_, and MgCl_2_ (0%, 5%, 10%, 15%, 20%) were added to the polymer solution, and hot-rolling recovery rate experiments were carried out as described above. The pH of all salt solutions was adjusted to 8.0 ± 0.1 using 0.1 M NaOH or HCl before testing, to mimic the typical alkaline drilling fluid environment.

#### 4.4.5. Contact Angle Measurement

The clay samples (untreated bentonite, bentonite treated with 1.5% HPEI, and bentonite treated with 1.5% HPEI-g-PDMAEMA) were compressed into pellets (diameter 25 mm, thickness 2 mm) under 10 MPa. The water contact angle was measured using a contact angle goniometer at room temperature. The water contact angle indicates the hydrophobicity of the clay gel surface after polymer treatment, which correlates with the inhibition performance against water invasion. Each sample was measured three times, and the average value was reported.

#### 4.4.6. Surface Tension Measurement

The surface tension of HPEI-g-PDMAEMA aqueous solutions at different concentrations (0.1, 0.5, 1.0, 1.5, 2.0 wt%) was measured using the Du Noüy ring method with a tensiometer (BZY-2, Shanghai Hengping Instrument Factory, Shanghai, China) at 25 °C. Each solution was allowed to stand for 10 min to reach equilibrium before measurement. The platinum ring was cleaned with deionized water and ethanol after each measurement. Each sample was measured three times, and the average value was reported. The critical micelle concentration (CMC) was determined as the concentration at which the surface tension stopped decreasing significantly with increasing concentration.

#### 4.4.7. API Filtration Test

The filtration properties of bentonite slurries (4 wt% bentonite) containing 1.5 wt% inhibitor were measured using an API low-pressure filter press (YN-1A, Qingdao Senxin) at room temperature under 0.69 MPa for 30 min. The filtrate volume was recorded, and the filter cake thickness was measured after the test.

## Figures and Tables

**Table 1:** Effect of feed ratio on product properties (pure water).

HPEI:DMAEMA	Grafting Ratio/%	M_w_ (g/mol)	Transmittance at 25 °C (%)	LCST/°C	Recovery at 150 °C/%
1:1	18.5	72,521	98.2	52	88.5
1:1.5	28.3	78,154	96.5	48	91.2
1:2	35.2	84,298	94.8	45	94.1
1:2.5	38.6	91,558	78.4	42	92.6
1:3	41.5	102,357	62.1	38	89.3

Note: All LCST values were measured in pure water at pH 8.0 (adjusted with 0.1 M NaOH/HCl), unless otherwise specified.

**Table 2:** Effect of AIBN dosage increase on product properties (pure water).

AIBN/HPEI	M_w_ (g/mol)	Grafting Ratio/%	PDI	Gelation	Recovery at 150 °C/%
1	125,243	24.8	2.85	present	86.3
1.6	102,259	31.2	2.41	slight	90.1
2.4	84,298	35.2	2.37	none	94.1
3	78,136	34.5	2.26	none	91.9
4	64,281	32.8	2.15	none	90.2

Note: All LCST values and performance data (recovery rates) in this table were measured at pH 8.0 (adjusted with 0.1 M NaOH/HCl), unless otherwise specified.

**Table 3:** Effect of different reaction temperatures on product properties (pure water).

Temperature/°C	M_w_ (g/mol)	Grafting Ratio/%	PDI	Gelation	Recovery at 150 °C/%
60	72,154	28.4	2.21	present	89.2
65	78,694	31.9	2.31	slight	91.5
70	84,298	35.2	2.37	none	94.1
75	92,551	36.9	2.42	none	92.4
80	108,496	37.6	2.56	none	88.6

Note: All LCST values and performance data (recovery rates) in this table were measured at pH 8.0 (adjusted with 0.1 M NaOH/HCl), unless otherwise specified.

**Table 4:** Effect of different reaction times on product properties (pure water).

Reaction Time/h	M_w_ (g/mol)	Grafting Ratio/%	PDI	Gelation	Recovery at 150 °C/%
6	68,204	24.6	2.18	none	86.5
8	76,815	30.7	2.25	none	90.2
10	84,298	35.2	2.37	none	94.1
12	96,115	36.9	2.48	none	93.2
14	102,489	37.5	2.61	slight	91.8

Note: All LCST values and performance data (recovery rates) in this table were measured at pH 8.0 (adjusted with 0.1 M NaOH/HCl), unless otherwise specified.

**Table 5:** Hydrodynamic particle size variation in HPEI-g-PDMAEMA under different pH conditions.

pH	Temperature/°C	Particle Size/nm
7	30	23.8
	55	354.6
8	30	29.4
	49	274.5
9	30	34.7
	44	201.8

**Table 6:** Cyclic thermoresponsive hydrodynamic diameter and PDI of HPEI-g-PDMAEMA (pH 8, 0.5 wt%) upon repeated heating (55 °C) and cooling (30 °C).

Cycle	Heated at 55 °C	Cooled to 30 °C (Recovered)
Initial	N/A	Dh = 29.4 nm, PDI = N/A
1	Dh = 274.5 ± 8.5 nm, PDI = 0.16 ± 0.02	Dh = 31.2 ± 1.5 nm, PDI = 0.09 ± 0.01
2	Dh = 268.3 ± 7.2 nm, PDI = 0.15 ± 0.02	Dh = 30.8 ± 1.3 nm, PDI = 0.08 ± 0.01
3	Dh = 265.1 ± 6.8 nm, PDI = 0.15 ± 0.02	Dh = 30.5 ± 1.1 nm, PDI = 0.09 ± 0.01

Note: Dh represents the hydrodynamic diameter determined by DLS.

**Table 7:** Hot-rolling recovery rate of shale at different salt concentrations (150 °C, 1.5 wt% HPEI and HPEI-g-PDMAEMA).

Salt Concentration/wt%			HPEI Recovery/%			HPEI-g-PDMAEMA Recovery/%		
NaCl	CaCl_2_	MgCl_2_	NaCl	CaCl_2_	MgCl_2_	NaCl	CaCl_2_	MgCl_2_
0	0	0	85.3	85.3	85.3	94.1	94.1	94.1
5	5	5	76.5	70.2	67.8	89.5	84.6	81.3
10	10	10	58.6	54.1	50.2	85.2	80.1	77.4
15	15	15	45.2	39.6	37.1	76.8	71.5	67.8
20	20	20	32.5	28.1	24.3	68.4	60.3	57.6

**Table 8:** Rheological properties of 20 wt% bentonite slurries treated with different inhibitors at 25 °C and 60 °C.

System	Temperature/°C	AV/mPa·s	PV/mPa·s	YP/Pa	YP/PV	GS_10_ s/Pa	GS_10min_/Pa
Blank	25	42.5	28.4	14.1	0.5	8.2	12.5
Blank	60	36.8	24.2	12.6	0.52	6.5	10.8
5 wt% KCl	25	38.2	26.5	11.7	0.44	6.8	10.2
5 wt% KCl	60	33	22.8	10.2	0.45	5.4	8.6
1.5 wt% D230	25	32.8	24.2	8.6	0.36	5.2	8
1.5 wt% D230	60	28.5	20.8	7.7	0.37	4.2	6.8
1.5 wt% HPEI	25	28.3	21.8	6.5	0.3	4.2	6.8
1.5 wt% HPEI	60	24.6	19	5.6	0.29	3.5	5.9
1.5 wt% HPEI-g-PDMAEMA	25	19.6	17.2	2.4	0.14	1.8	3.2
1.5 wt% HPEI-g-PDMAEMA	60	16.8	14.8	2	0.14	1.5	2.8

Note: AV = θ_600_/2; PV = θ_600_ − θ_300_; YP = θ_300_ − PV; GS_10s_ and GS_10min_ were measured after 10 s and 10 min of rest, respectively. All measurements were performed at pH 8.0 ± 0.1.

**Table 9:** API filtration performance of bentonite slurries treated with different inhibitors.

Sample	Filtrate Volume (mL)	Filter Cake Thickness (mm)
Blank	26.8 ± 0.5	3.2 ± 0.2
+1.5 wt% HPEI	18.5 ± 0.4	2.5 ± 0.1
+1.5 wt% HPEI-g-PDMAEMA	12.3 ± 0.3	1.8 ± 0.1

**Table 10:** Comparison of inhibition mechanisms between HPEI-g-PDMAEMA and HPEI-PNIPAM-PO (literature).

Feature	HPEI + PNIPAM + PO (Terpolymer)	HPEI + PDMAEMA (Binary)
Molecular composition	HPEI + PNIPAM (amide) + PO	DMAEMA (tertiary amine)
LCST (pH 8~9)	~32 °C	~45~48 °C
Thermosensitive trigger timing	Collapses at the surface or a shallow depth (premature consumption)	Triggered only at bottomhole high temperatures (temperature-targeted)
Primary anchoring force	Hydrogen bonds	Electrostatic attraction
Hydrophobic barrier source	PO (permanent) + PNIPAM collapse (temporary)	DMAEMA collapse only (temporary hydrophobic)
Inhibition mechanism hierarchy	Hydrogen bonding anchoring + premature thermosensitive plugging + permanent hydrophobicity	Electrostatic anchoring + bottomhole thermosensitive plugging (dual synergy)
Molecular design feature	Requires grafting of two different side chains; more complex synthesis	Grafting of a single monomer, simple synthesis, good water solubility
Application advantage	Stronger hydrophobicity, but premature phase transition and weak anchoring	Smart thermosensitive window, strong anchoring, effective over a wide temperature range

**Table 11:** Mineral composition of shale cuttings and clay.

Category	Composition	Content (wt%)
Mineralogical	Quartz	38.5
	Potassium feldspar	5.2
	Plagioclase	12.8
	Calcite	6.5
	Dolomite	3.2
	Pyrite	2.8
Clay minerals	Illite	48.5
	Illite/smectite mixed layer	32.6
	Chlorites	12.4
	Kaolinite	6.5

## Data Availability

The data that support the findings of this study are available from the corresponding author upon reasonable request.
